# Research on the Output Characteristics of Energy Conversion Elements under External Excitation

**DOI:** 10.3390/mi14030549

**Published:** 2023-02-26

**Authors:** Yun Zhang, Zonglin Xiao, Lan Liu, Wei Ren, Wei Liu, Yanjie Gou, Xiaoming Ren

**Affiliations:** 1 School of Mechano-Electronic Engineering, Xidian University, Xi’an 710071, China; 2 Science and Technology on Applied Physical Chemistry Laboratory, Shaanxi Applied Physics and Chemistry Research Institute, Xi’an 710061, China

**Keywords:** initiating explosive device, energy conversion element, different shape, external excitation, temperature and current field

## Abstract

Initiating explosive (IE) devices are widely used in the aerospace, resource mining, and basic industries and other fields. With the improvement in processing technology, IE devices are developing towards miniaturization and intelligence. As an important component of the energy conversion of IE devices, the output characteristics of micro energy conversion (EC) elements directly affect the ignition performance of IE devices. Hence, this paper researches the output characteristics of EC elements under external excitation. Firstly, the fabrication process of the EC element is introduced, and the finite element analysis model of the temperature field is deduced. Secondly, the simulation model of the output characteristics of the EC element is constructed, the validity of the model is verified through experiments, and the basic characteristic parameters of the EC element are determined. Finally, four shapes of EC element structures are designed, the corresponding output characteristics under constant current excitation are analyzed, and the temperature and current field distributions of the EC elements with different shapes are given. The experimental and simulation results show the effectiveness of the analysis results in this paper, and the influence of different shapes on the insensitivity of EC elements is given through comparative analysis, which provides support for the design of micro structure EC elements.

## 1. Introduction

As a key component of initiating explosive (IE) devices for energy conversion, energy conversion (EC) elements have always been the focus of research in IE devices, and directly affect the comprehensive performance of an IE system [1,2]. In modern environments, the application scenario of explosives has become more complex [3]. With the improvement in ignition and safety standards, higher requirements are being put forward for the ignition and insensitivity performance of explosives. For instance, the standard for insensitive IE devices is usually that the ignition will not occur after continuous excitation for five minutes under direct-current (DC) 1 A or 1 W power. However, due to the limitations of monitoring technology and the one-time use characteristics of IE devices, it is difficult to carry out insensitivity experiments for the EC elements, so it is necessary to enrich the analysis methods of the output characteristics of the EC elements.

Enhancing the output characteristics of the EC elements is the main way to improve the ignition and insensitivity of IE devices [1,4]. To optimize the output performance of the EC elements, micro electro-mechanical systems (MEMS) technology has been introduced into IE devices, leading to the generation of high-performance micro EC elements [5,6,7]. Micro EC element materials mainly include semiconductor bridges [8,9,10], metal material membrane bridges such as nickel–chromiums alloy [11,12], tantalum nitride (TaN) [13,14], and composite materials [15,16,17]. Among these materials, TaN is widely used because of its high stability at high temperatures, antioxidant properties, corrosion resistance, and low cost of manufacturing [18,19].

Furthermore, besides the fact that different materials directly affect the performance of EC elements, different microstructures have different Joule effects [20,21], which means improving the three-dimensional topography of EC elements can also effectively improve the output characteristics. Therefore, the influence of shape on the characteristics of EC elements has long been a focus of research [22]. The temperature characteristic is the most critical index of the EC elements, and temperature probes are usually used to acquire the output characteristics of EC elements. However, with the development of EC elements towards smaller and more complex shapes, the installation space and response accuracy of the current probes cannot meet these needs. Therefore, a reasonable and accurate method for acquiring and analyzing the characteristics of EC elements should be presented.

Multi-physical field simulation is a reliable research method, which can reflect the characteristics and guide the design of EC elements with complex shape [23,24]. In view of this, this paper studies the output characteristics of TaN EC elements prepared using MEMS technology. The main scientific contribution of this paper is to propose a multi-physical field simulation method for the analysis of the output characteristics of EC elements, and verify the validity of the constructed model through experiments. Based on the proposed method, the influence law of the shape on TaN EC elements is analyzed and summarized.

The paper is structured into four sections. Following the introduction, the fabrication process and conversion analysis of EC elements are introduced in Section 2. Section 3 verifies the validity of the simulation model by comparing experimental and simulation results, and gives the output characteristics under the excitation of ignition analysis. In Section 4, to study the influence of shape on the insensitivity of EC elements, four types of EC element shapes are designed and analyzed. Finally, the conclusions are given in Section 5.

## 2. Fabrication Process and Conversion Analysis of EC Elements

### 2.1. Fabrication Process

**Figure 1:**
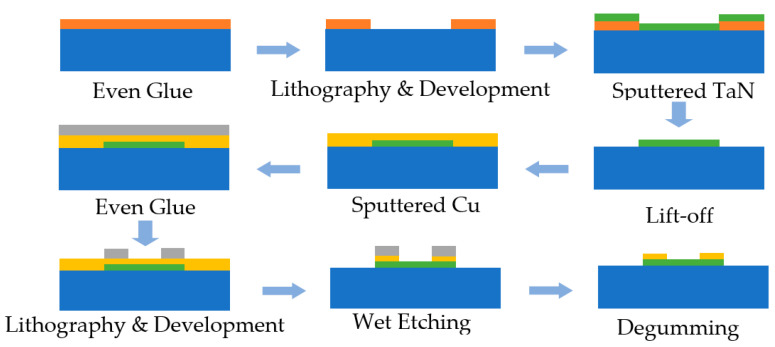
Fabrication of TaN bridge film EC element.

The fabrication process of MEMS EC elements is shown in Figure 1, including bridge film sputtering, bridge area patterning, pad film sputtering and pad patterning. The patterning method included etching and stripping. For the TaN bridge area, because etching requires the use of corrosive reagents, in order to ensure the quality of the bridge area graphics, the TaN bridge used stripping technology. For copper pads, etching technology was used to efficiently form the pad structure. The overall process flow is as follows:

1. The photoresist was coated on the substrate, and the photoresist became a mask with a specific pattern after photolithography and development;
2. The TaN film was deposited by magnetron sputtering, and the photoresist and the film material on it were peeled off with reagents, leaving only the designed film pattern on the substrate;
3. The copper pad was deposited by magnetron sputtering technology, coated with photoresist, and the photoresist in the pad area was protected by photolithography;
4. The reagent was used to corrode the metal film area not protected by the photoresist, and then the brazed pad pattern was formed.

**Figure 2:**
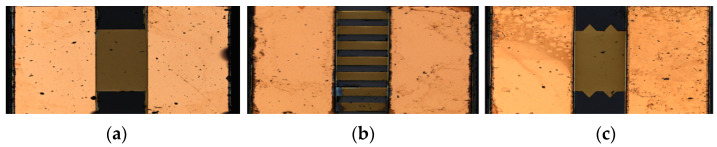
Fabricated Samples of MEMS EC elements. (**a**) Rectangular shape; (**b**) Strip shape; (**c**) Angular shape.

Through the above fabrication process, TaN EC elements with the different shapes shown in Figure 2 can be obtained.

### 2.2. Theoretical Analysis of Energy Conversion

The mechanism of the function of EC elements is the Joule thermal effect, that is, when the current passes through the conductor, electrical energy is converted into heat energy [25], which was discovered by the British physicist Joule. In the Joule effect, the relationship between electric energy and thermal energy is summarized in the Joule law, and its mathematical expression is as follows:

(1) $$Q=I^{2}Rt$$

For regular shapes, the resistance can be calculated using the following formula:

(2) $$R=\frac{L}{\sigma A}$$

where *σ* is the conductivity of the conductor material, *L* is the length of the conductor and *A* is the cross-sectional area of the conductor through which the current passes.

The above Joule effect formula and resistance calculation formula are applicable to the overall analysis of conductors with a regular shape. For the complex shape of the EC element, more in-depth study was required. Due to the complex shape, the electric field intensity of each part is different, which leads to the current density of each part and the heat generation of Joule heating also being different.

The relationship between the electric field and current density is:

(3) $$\vec{E}=-\nabla \varphi$$

(4) $$\vec{J}=\sigma \vec{E}$$

(5) $$\nabla \cdot \vec{J}=\rho_{e}$$

where $\vec{E}$ is electric field (V/m), φ is electric potential (V), $\vec{J}$ is electric current density (A/m^2^), and $\rho_{e}$ is charge density (C/m^3^).

Here, a constant current is applied to the EC element, $\rho_{e}=0$. The heat generation rate of Joule effect is:

(6) $${\dot{Q}}_{J}=\frac{{\left|\vec{J}\right|}^{2}}{\sigma }$$

There is a temperature field in the object. The heat conduction problem of the energy converter is unsteady heat conduction, and the temperature field changes with time:

(7) t=f(x,y,z,τ)

In addition to the heat generated by each part of the energy exchanger, the heat conduction is also generated by the temperature difference. It can be described by a uniform heat conduction control equation:

(8) $$\rho c\frac{\partial t}{\partial \tau }=\frac{\partial }{\partial x}\left(\lambda \frac{\partial t}{\partial x}\right)+\frac{\partial }{\partial y}\left(\lambda \frac{\partial t}{\partial y}\right)+\frac{\partial }{\partial z}\left(\lambda \frac{\partial t}{\partial z}\right)+{\dot{Q}}_{J}$$

where ρ is density(kg/m^3^), c is specific heat capacity (J/(kg·°C)), and λ is thermal conductivity (W/(m·°C)).

Initial and boundary conditions are required to fully describe the unstable heat conduction problem. For the EC element, the initial temperature is uniform. The initial conditions are:

(9) $$t\left(x,y,z,0\right)=t_{0}$$

The boundary conditions are generally divided into three categories, the third category of which is as follows. The surface heat transfer coefficient *h* between the object on the boundary and the surrounding fluid and the temperature of the surrounding fluid $t_{f}$ are specified:

(10) $$-\lambda \left(\frac{\partial t}{\partial n}\right)=h\left(t-t_{f}\right)$$

The transient temperature change in an EC element under external excitation is calculated by solving the finite element equation for transient thermal analysis as follows:

(11) $$\left[C\right]\left\{\dot{T}\right\}+\left[K\right]\left\{T\right\}=\left\{Q\right\}$$

## 3. Numerical Verification and Analysis of Ignition Characteristics

### 3.1. Material Parameters and Test System Configuration

**Figure 3:**
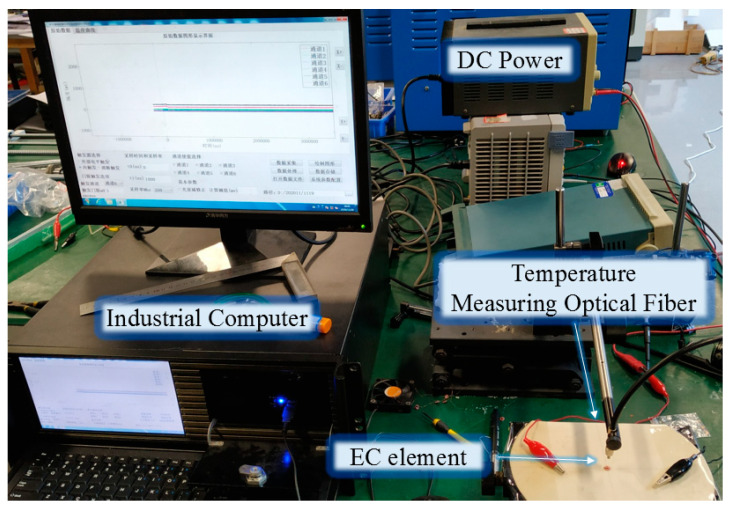
Transient high temperature testing system.

In this study, multi-physical field simulation was used to analyze the output characteristics of EC elements. In order to accurately obtain the analysis parameters of EC elements and verify the effectiveness of the theoretical analysis, the experimental verification was carried out in an atmospheric environment, and the experimental platform is shown in Figure 3. The tested EC element was fixed on the optical lifting platform. The Tektronix PWS4305 programmable power supply, LINI-T UTP3704 adjustable power supply, 33 uF capacitor, and a mercury switch were used to realize the excitation loading of the EC element.

The temperature of the EC element was measured by a transient pyrometer. The transient optical pyrometer used photoelectric conversion technology to test the changing process of the radiance of the EC element. Then, based on the Planck thermal radiation theory, the radiance of the EC element was compared with that of the standard light source, so as to measure the temperature of the EC element.

In the experiment, through the guidance of an auxiliary light source, the temperature measuring optical fiber was aligned with the center of the EC element, which is also the part with the highest temperature. The response wavelength of the transient pyrometer was 0.90 μm~1.62 μm, the temperature measurement range was 900 K~3500 K, the response time was not more than 1μs, the measurement error was less than 2%, and the high sampling rate was 250 MHz.

The material parameters of the TaN EC elements are shown in Table 1. The relationship between the resistance and temperature is called the temperature characteristic of resistance (TCR). According to the experimental results and inverse approximation analysis, the TCR relationship of TaN was obtained, as shown in Table 1, where the unit of T was K.

**Figure 4:**
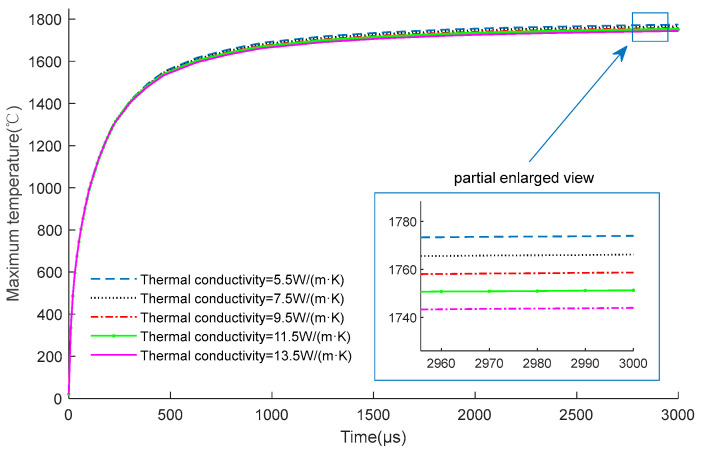
Comparison of the temperature of the EC element (0.2 mm × 0.1 mm × 0.9μm) with different thermal conductivity under 6V constant voltage excitation.

It should be noted that the thermal conductivity of TaN will also change with temperature. Figure 4 shows the temperature change curves of the EC element under different thermal conductivity conditions. It can be seen that the thermal conductivity has little effect on the temperature of the EC element. Therefore, the thermal conductivity of TaN is regarded as a constant value in Table 1.

### 3.2. Output Characteristics under the Excitation of Ignition Analysis

**Figure 5:**
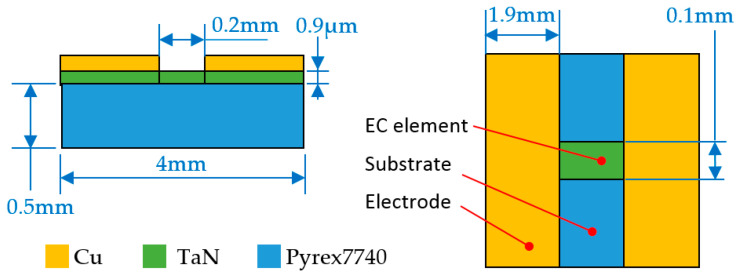
Three dimensions and material of EC element under ignition analysis.

In the analysis of ignition excitation, the temperature change curve of the EC element reaching the detonation point was mainly studied, with the purpose of enabling the EC element to reach the detonation condition quickly. The three dimensions of an EC element of TaN material are shown in Figure 5. The length, width and thickness of the EC element were 0.2 mm, 0.1 mm, and 0.9 μm, respectively, with a resistance of 10 Ω. Pyrex7740 glass was used as the substrate of the energy exchange element, and its thermal conductivity is small, which was conducive to a rapid temperature rise.

**Figure 6:**
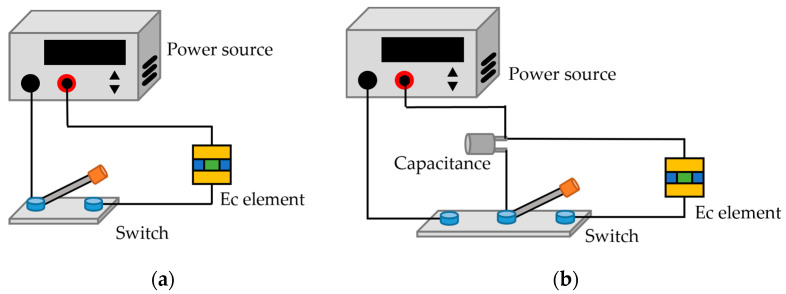
External excitation circuit. (**a**) Constant voltage excitation; (**b**) Capacitor discharge excitation.

During the analysis of ignition excitation, capacitor discharge and constant voltage excitation were used to realize the energy supply of the EC element. The constant voltage excitation circuit is shown in Figure 6a, and the excitation voltage was 6 V. Figure 6b is a capacitor discharge excitation circuit; the capacitor charging voltage and the capacitance value were 20 v and 33 μF, respectively.

In order to analyze the effectiveness of the theoretical analysis of energy conversion, the conversion performance of the EC element, shown in Figure 5, was studied through theoretical analysis and the commercial software COMSOL. According to the physical field involved in the model of the EC element, the software adjusts the mesh based on the geometric structure, refines the cell size and generates a tetrahedral mesh. Moreover, the grid near the corner of the EC element is dense, while the grid at the other parts is sparse.

**Figure 7:**
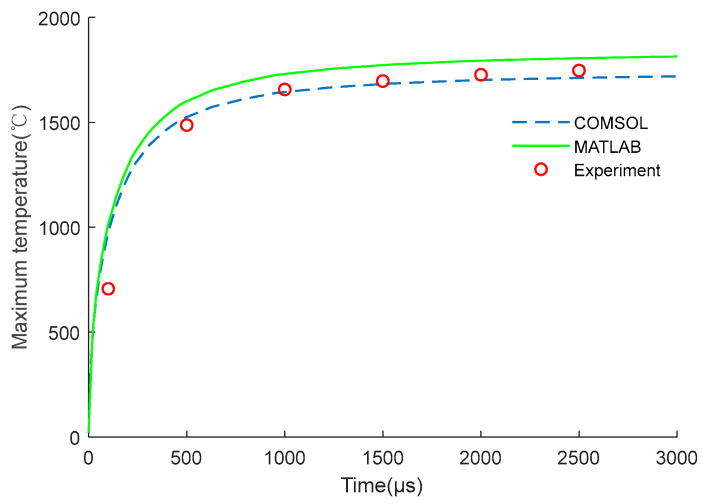
Simulation and experimental results under constant voltage excitation.

**Figure 8:**
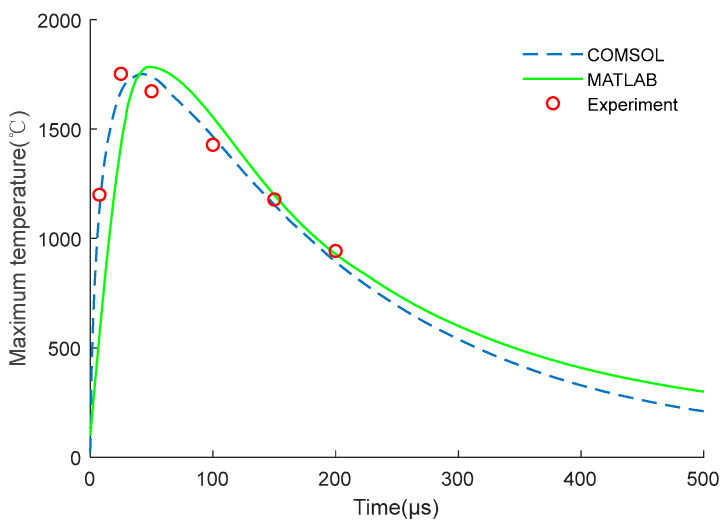
Simulation and experimental data under capacitive discharge.

In the calculation model, the surface nodes at both ends of the brazed pad were galvanically coupled. The influence of air convection and thermal radiation on the heat distribution was also considered. Here, set the air thermal convection coefficient of all external surfaces of the EC element model was set as 10 W/(m^2^ °C) and the ambient temperature was 293.15 K. Through the analysis of COMSOL software, the maximum temperature distribution of the EC element can be obtained. Meanwhile, the temperature distribution results could also be obtained by building the numerical model of the EC element on MATLAB through Equation (11). In addition, the temperature could also be measured through the experimental platform in Figure 3. The comparisons between the numerical calculation results and experimental data are shown in Figure 7 and Figure 8.

It can be seen from Figure 7 that the simulation results were consistent with the experimental results. After the excitation was applied, the temperature obtained by numerical calculation and COMSOL software was 1805.8 °C and 1712.3 °C, respectively; the experimental temperature was 1747.1 °C; and the simulation error was 3.36% and 1.99%, respectively.

Figure 8 also shows the simulation and experimental data under capacitive discharge, the two datasets are basically consistent. The reasons for the difference between MATLAB and COMSOL may be that the element division in the MATLAB simulation is simpler, while the COMSOL mesh division is more accurate. Moreover, the employed material parameters are slightly different between the actual application and the simulation, and there are also deviations in the experimental measurement process, which induce errors between the experiment and simulation.

As shown in Figure 7 and Figure 8, the experiment proves that the numerical simulation results are reliable, the design and research process of the EC element can be appropriately simplified and replaced by numerical simulation in the subsequent study.

## 4. Analysis of Insensitivity Characteristic under Different Shapes

### 4.1. Output Characteristics under the Excitation of Insensitivity Analysis

**Figure 9:**
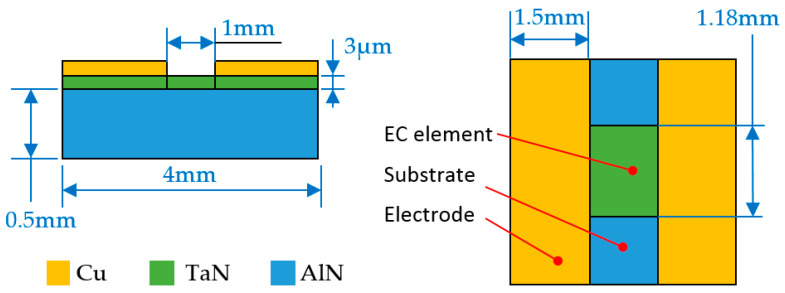
Three dimensions and material of EC element under insensitivity analysis.

In the analysis of insensitive excitation, the main focus was whether the temperature of the EC element was within the safe threshold range under long-term constant excitation. The excitation circuit of insensitive analysis was the same as that shown in Figure 6a, and aluminum nitride (AlN) was used as the substrate material; its thermal conductivity was 180 W/(m·°C). The AlN substrate is fixed on a circular epoxy resin plate with a diameter of 19 mm and a thickness of 2.5 mm, and the thermal conductivity of the resin plate is 1.2 W/(m·°C). The common structural form of the EC element is shown in Figure 9. The length, width and thickness of the EC element were 1 mm, 1.18 mm, and 3 μm, respectively, and the resistance value was 1 Ω. The physical image of EC element is shown in Figure 2a.

**Figure 10:**
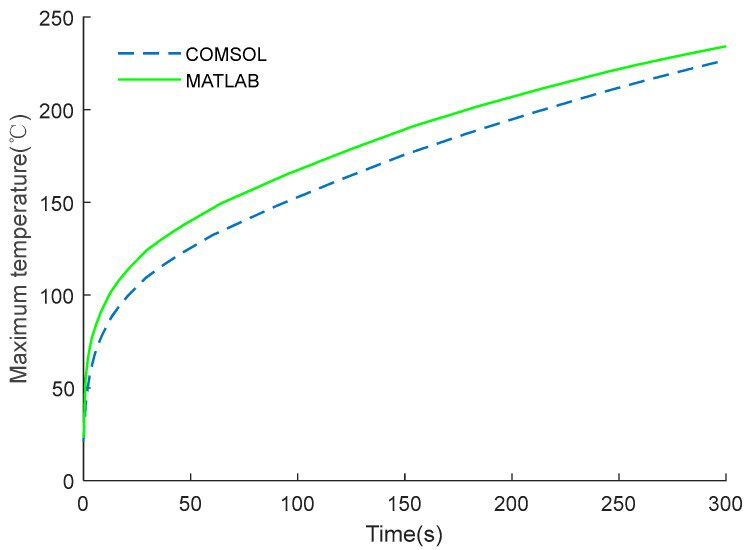
Calculation results under constant voltage excitation.

The conversion performance of the EC element shown in Figure 9 was studied through theoretical analysis and the commercial software COMSOL. According to the national standard of GJB 344A–2005 [26], under the temperature conditions of 21 ± 3 °C and 107 ± 3 °C, the class A insensitivity IE device should be able to ensure that the EC element does not ignite within five minutes. Therefore, the simulation time here is set to 300 s. It can be seen from Figure 10 that the MATLAB results are in good agreement with the COMSOL’s calculation results. After applying constant current excitation for 300 s, the calculated temperature was 249.1 °C and 226.7 °C, respectively. Since the temperature is not within the measurement range of the pyrometer, it is obtained by conventional methods, and the measured temperature was about 230.6 °C in the experiment. The analysis error of theoretical analysis and the COMSOL was 8.02% and 1.69%, respectively. 

In addition, it should be noted that in the insensitive analysis, compared with Figure 5, the substrate material in Figure 9 was changed from 7740 glass to AlN. The thermal conductivity of the substrate, of which the bridge is made, plays a main role in the thermal flow, and directly effects the temperature characteristic.

Due to the limitations of the installation space, the response accuracy of the temperature sensor and the one-time use characteristics of EC elements, it is difficult to conduct a large number of insensitive experiments on complex-shape EC elements under constant current excitation, which cannot support the comparative analysis of EC element characteristics. Therefore, in the subsequent study, to facilitate the acquisition of the insensitivity characteristics of the EC element with complex micro shapes, the analysis will be mainly based on the commercial software.

**Figure 11:**
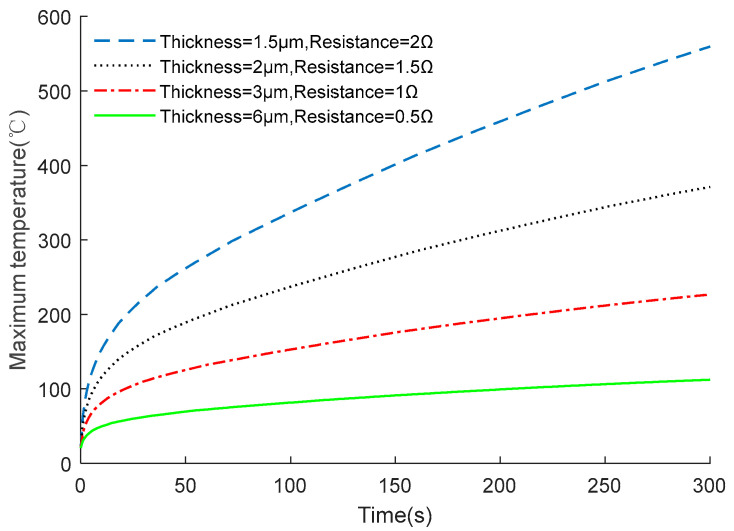
Output characteristics of EC elements with different resistance values.

The resistance value of the EC element in Figure 9 is 1 Ω. To analyze the characteristics of the EC element with different resistance values under constant current excitation, the resistance value was adjusted by changing the thickness of the EC element, and then the corresponding output temperature is shown in Figure 11.

As shown in Figure 11, resistance value has a great influence on the characteristics of the EC element. However, the GJB 344A-2005 national standard requires that the minimum current and power of the DC excitation applied to the EC element are 1 A and 1 W, respectively. Hence, it is necessary to research and design an optimal shape of the EC element to further improve its insensitivity performance, with a fixed resistance value of 1 Ω.

**Figure 12:**
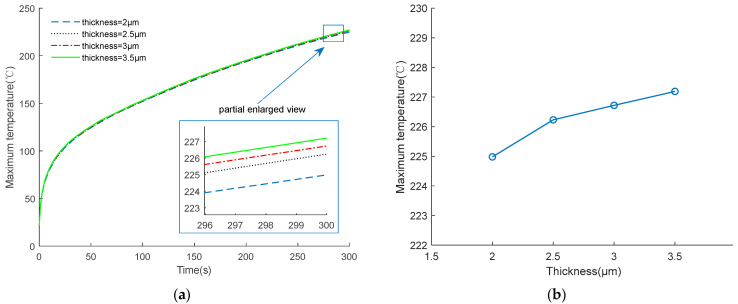
Temperature changing of EC element under different thicknesses. (**a**) Temperature change curve; (**b**) Maximum temperature at 300 s.

To study the influence of various shape parameters on the output characteristic, the EC element in Figure 9 was taken as the research object, keeping the resistance unchanged, and exploring the influence of the thickness on the temperature of the EC element by changing the width of the bridge zone. The relationship curve between the thickness and temperature is shown in Figure 12.

**Figure 13:**
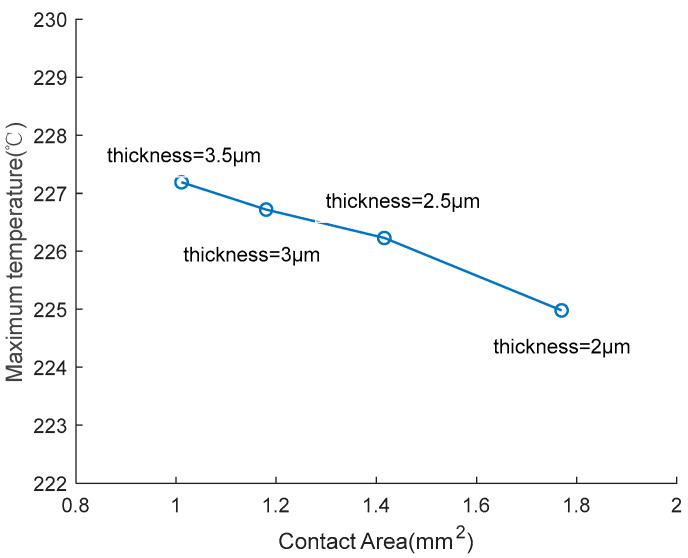
Temperature change of EC element under different contact areas.

Figure 12 shows that as the thickness decreases, the maximum temperature of the EC element also decreases. The reasons for this phenomenon are as follows, in order to keep the resistance at 1 Ω when the thickness decreases, the width needs to increase, which leads to an increase in surface area, which means the heat exchange capacity with the substrate is enhanced. Therefore, as shown in Figure 13, the smaller the thickness, the larger the surface area, which leads to a lower temperature, and represents a better insensitive characteristic.

### 4.2. The Influence of Different Shapes on the Output Characteristics

**Figure 14:**
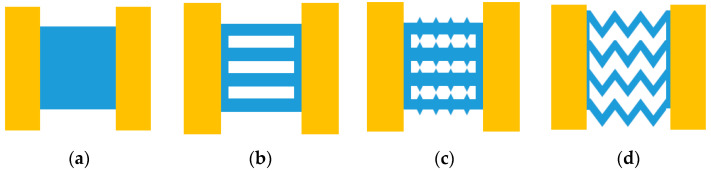
Four types of EC elements. (**a**) shape A; (**b**) shape B; (**c**) shape C; (**d**) shape D.

To further study the influence of various shapes on the characteristics of the EC element, four types of EC element were designed and they are shown in Figure 14. Among the four shapes, the three-dimensional size and material of shape A was same as the EC elements shown in Figure 9.

**Figure 15:**
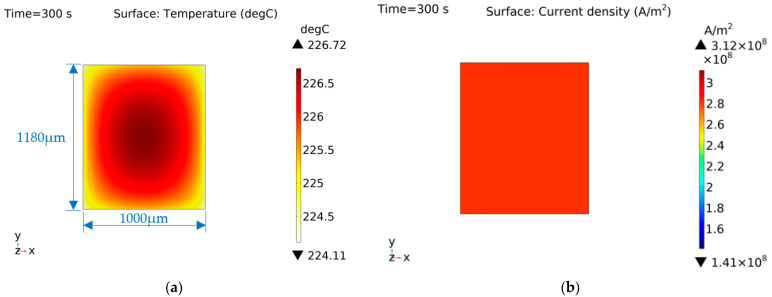
Characteristic distribution of shape A with a constant current (1 A) excitation. (**a**) Temperature distribution; (**b**) Current density distribution.

**Figure 16:**
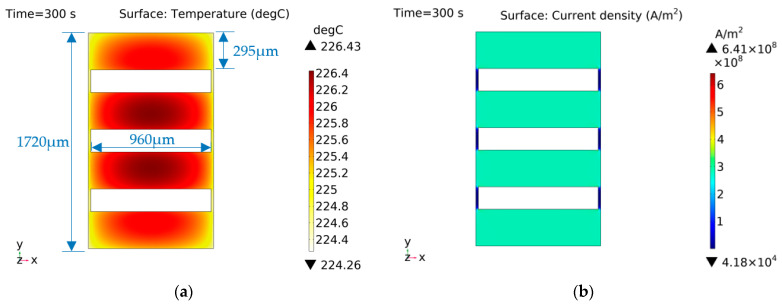
Characteristic distribution of shape B with a constant current (1 A) excitation. (**a**) Temperature distribution; (**b**) current density distribution.

**Figure 17:**
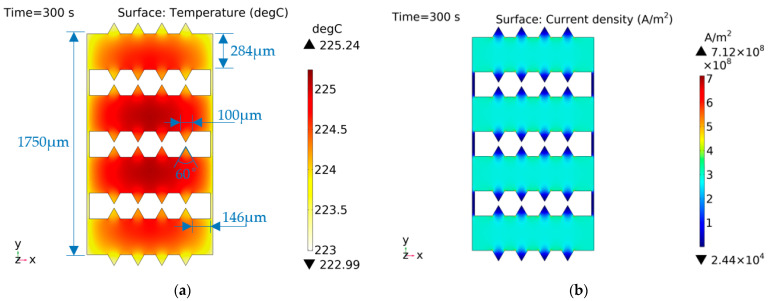
Characteristic distribution of shape C with a constant current (1 A) excitation. (**a**) Temperature distribution; (**b**) current density distribution.

**Figure 18:**
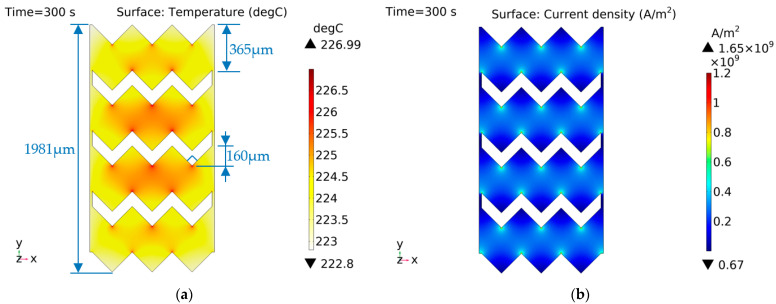
Characteristic distribution of shape D with a constant current (1 A) excitation. (**a**) Temperature distribution; (**b**) current density distribution.

The length of each EC element of different shape in Figure 14 was set as 1 mm, and the thickness was 3 μm, and the only the width of the EC element was adjusted to ensure that the resistance was always 1 Ω in the different shapes. Through the analysis of the four shapes, the influence of the shapes on the characteristics of the EC element are described in Figure 15, Figure 16, Figure 17 and Figure 18.

Figure 15b shows that the current density distribution of shape A was uniform, so the heat generation of each part of the EC element was equal. The temperature distribution shows that the temperature of shape A at the edge was small, because the two flanks of the EC element were connected with the copper electrodes and had strong heat conduction. Moreover, since the edge of the EC element conducted heat to the base more quickly, the central temperature of shape A was the highest.

The characteristic distribution of shape B is shown in Figure 16. This shape had voids in the bridge area to enhance heat dissipation at the center of the EC element. The overall current density of the EC element was the same, except for the area close to both sides of the brazed pad.

As shown in Figure 17, shape C had sharp angles to branches on the basis of shape B, and the temperature was slightly lower than that of shape B. The current density distribution shows that the current density at these angles was significantly lower than that of the branches, indicating that the temperature rise at angles is more dependent on the heat conduction of other parts with higher temperatures.

The characteristic distribution of shape D is shown in Figure 18. Shape D used a wavy EC element, and there was a large difference between the inward and outward sharp current density of this shape. According to Figure 18b, it can be seen that the inward sharp current density is higher, which generates more heat and more concentrated temperature distribution.

In Figure 15, Figure 16, Figure 17 and Figure 18 the surface area of the four shapes of EC elements are 1.18 mm^2^, 1.2 mm^2^, 1.17 mm^2^ and 1.48 mm^2^, respectively. According to Figure 13, based on the typical dependence for heat flow, the larger the surface area and the larger the contact area with the substrate, the faster the heat flow. Hence, the temperature of shape D should be the lowest.

**Figure 19:**
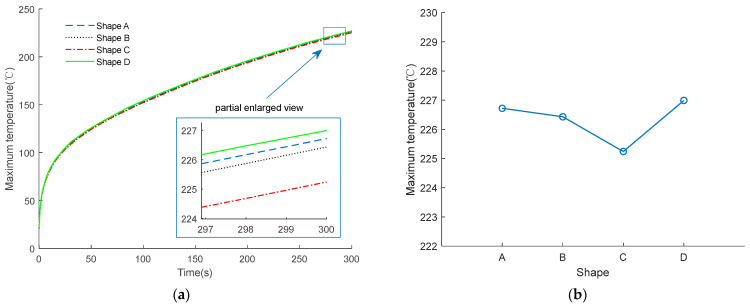
Temperature comparison of four shapes at 1 A constant current excitation. (**a**) Temperature change curve; (**b**) maximum temperature at 300 s.

However, as shown in Figure 19, through the comparison and analysis of the proposed different shapes, shape C has the lowest temperature, while shape D has the highest temperature. The reasons for this phenomenon are as follows:

The current density will change greatly at the position where the shape of the EC element changes significantly. The position with the higher current density will produce more Joule heat, and lead to a higher temperature than in other parts. Hence, although shape D has the largest contact surface area with the substrate, due to its complex shape, the highest temperature occurs at the highest current density area among the four shapes. As for shape C, there are also structural mutations; the current density at the sharp corner is low, while that at the junction of the sharp corner and the strip structure is high, but this position is not conducive to heat focusing, and the temperature is the lowest, which finally shows it has the best insensitivity among the four EC element shapes with a resistance of 1 Ω under 1 A current excitation. 

Comparing the current density and temperature distribution of the four shapes, the following can be concluded:

1. Under the same conditions, the smaller the resistance of the energy conversion element, the lower the temperature.
2. When the resistance value is constant, the smaller the thickness of the EC element, the larger the contact area with substrate and the lower the temperature.
3. When the resistance value is constant, the current density of the inner corner is high and that of the outer corner is low. The peak position of the inner corner easily forms a high temperature region, while the outer corner shows the opposite trend.
4. It is not easy to form a high temperature at the junction of the EC elementand the electrode, while the temperature is higher in the central region.

In all, considering that sharp corners have obvious impacts on the performance of EC elements, specific feature structures should be reasonably designed and used to optimize the characteristics of EC elements. Moreover, since microstructural characteristics in the shapes shown in Figure 14 are difficult to be detected in actual tests, but this defect can be remedied by multi-physical field simulation analysis.

## 5. Conclusions

In this paper, the output characteristics of EC elements under external excitation were analyzed. The following conclusions can be drawn from the experiments and simulation analysis of different shapes of TaN EC elements:

1. The fabrication process and conversion analysis of TaN EC elements was studied. The output characteristics under the excitation of ignition analysis were obtained, and the experimental and simulation results show the effectiveness of the introduced theoretical analysis of energy conversion.
2. The resistance value of EC elements and the thermal conductivity of the substrate have a significant impact on the output characteristics. If the resistance value and thermal conductivity is constant, the shape of the EC element will also affect the temperature distribution. In addition, changing the contact area of the EC element will also affect its characteristics. The larger the contact area, the lower the temperature of the EC element.
3. Through multi-physical field analysis, more details of temperature and current density distribution are displayed, and it is observed that current density will change greatly at the position where the shape of the EC element changes dramatically, thus directly affecting the temperature distribution of the EC element by the Joule effect, and then affecting the insensitivity of EC elements.

Due to the inconsistency of the material properties and process in the fabrication of EC elements, the employed material parameters are slightly different between the actual application and the simulation, and there are also deviations in the experimental measurement process, which will affect the accuracy of the constructed analytical model. In addition, the practical application of EC elements is limited by field environments, so the specific design of EC elements should integrate temperature, excitation, and installation conditions, among other factors.

## Figures and Tables

**Table 1:** Material parameters of the bridge area of the TaN EC element.

Parameter	Value	Unit
Density	9540	kg/m^3^
Specific heat capacity	260	J/(kg·°C)
Thermal conductivity	9.54	W/(m·°C)
Convective heat transfer coefficient	10	W/(m^2^·°C)
Resistivity	0.00485T + 2.12	μΩ·m

## Data Availability

Not applicable.
